# Amandys® pyrocarbon interposition wrist arthroplasty: a systematic review of clinical outcomes and complications

**DOI:** 10.1016/j.jham.2026.100442

**Published:** 2026-02-28

**Authors:** Francesco Smeraglia, Lucian Lior Marcovici, Morena Anna Basso, Alessio Bernasconi, Andrea Cozzolino, Giovanni Balato

**Affiliations:** a Department of Public Health, Trauma and Orthopaedics, University of Naples “Federico II”, Naples, Italy; b Unit of Hand and Microsurgery, Ospedale Israelitico di Roma, Rome, Italy

**Keywords:** Wrist arthroplasty, Pyrocarbon, Amandys, Wrist salvage procedures, Proximal row carpectomy, Complications

## Abstract

**Background:**

Pyrocarbon interposition arthroplasty (Amandys®) has been proposed as a motion-preserving salvage procedure for advanced wrist degeneration. However, its clinical outcomes, complication profile, and role within the contemporary wrist salvage algorithm remain incompletely defined.

**Objective:**

To systematically review the clinical outcomes, complications, and revision patterns associated with Amandys® pyrocarbon interposition wrist arthroplasty, without performing a direct comparative analysis with other wrist salvage procedures, and to clarify its role among current treatment options.

**Materials and methods:**

A systematic review of the literature was conducted using PubMed, Scopus, and Web of Science databases. The search term “Amandys” was used without additional filters. Clinical studies reporting outcomes of Amandys® wrist arthroplasty were included. Data extraction focused on study characteristics, clinical outcomes, complications, and revision procedures. Methodological quality was assessed using the Coleman Methodology Score.

**Results:**

Ten clinical studies met the inclusion criteria, comprising a total of 224 wrists treated with Amandys® interposition arthroplasty. Pain reduction and improvement in patient-reported outcome measures were consistently reported across studies, with preservation of wrist range of motion. Complication rates were substantial, with approximately one fifth of wrists experiencing postoperative complications and nearly one fifth requiring reoperation. Most secondary procedures were related to early mechanical instability and consisted of technically oriented revisions rather than implant removal. Conversion to total wrist arthrodesis occurred less frequently. The overall methodological quality of the included studies was moderate.

**Conclusion:**

Amandys® pyrocarbon interposition arthroplasty provides clinical improvement and preserves wrist motion but is associated with a relevant and less predictable rate of complications and reoperations. When feasible, proximal row carpectomy remains the preferred motion-preserving procedure. Amandys® arthroplasty should be regarded as a highly selective salvage option, particularly in patients unsuitable for proximal row carpectomy–based solutions, with careful patient selection being essential to optimize outcomes.

## 1 Introduction

Degenerative and post-traumatic destruction of the radiocarpal and midcarpal joints, including scapholunate and scaphoid nonunion advanced collapse patterns, remains a therapeutic challenge when pain and functional limitation persist despite non-operative treatment. Motion-preserving salvage procedures such as proximal row carpectomy and partial wrist fusion are commonly used, but their indications may be limited in advanced or complex cases, particularly after failed previous surgery.

Pyrocarbon interposition arthroplasty using the Amandys® (Wright Medical–Tornier, Bioprofile, Grenoble, France) implant was introduced as a motion-preserving option for extensive wrist joint destruction in selected, well-aligned wrists with competent capsuloligamentous structures.^1^ Early clinical series reported improvements in pain and patient-reported outcomes with preservation of wrist mobility, both as a primary procedure and as a salvage option after failed wrist surgery 2, 3, 4. Subsequent studies extended the indications to inflammatory conditions, including rheumatoid arthritis, with encouraging mid-term functional results.^5^

More recent investigations with larger cohorts and longer follow-up suggested that most complications occur early after surgery, while clinical and functional outcomes may remain stable over time in patients without early failure.^6^ However, other reports and letters to the editor have highlighted a non-negligible rate of early instability, dislocation, and revision, raising concerns regarding patient selection and surgical technique.^7^^,^^8^

Pyrocarbon has been proposed for unconstrained interposition arthroplasty because of its modulus of elasticity closer to that of cortical bone compared with metallic implants, potentially allowing more physiological load transmission and reduced stress shielding.

Given the limited number of studies, small sample sizes, and heterogeneity in indications and outcome reporting, a comprehensive synthesis of the available evidence is warranted.

The aim of this systematic review is to summarize the current clinical evidence on Amandys® pyrocarbon interposition wrist arthroplasty, focusing on indications, functional outcomes, complications, and revision rates.

Although the number of published clinical studies on Amandys® arthroplasty is limited, this reflects the selective indications and technically demanding nature of the procedure rather than a lack of clinical relevance. In this context, a systematic review remains valuable to critically appraise indications, failure patterns, and decision-making strategies for a motion-preserving salvage technique used in highly selected cases.

## 2 Methods

This systematic review was conducted in accordance with the Preferred Reporting Items for Systematic Reviews and Meta-Analyses (PRISMA) guidelines.

A comprehensive literature search was performed using three electronic databases: PubMed, Scopus, and Web of Science. Given the uniqueness of the implant name, the search strategy was deliberately restricted to the single keyword “Amandys”, which uniquely identifies the pyrocarbon interposition wrist implant and does not overlap with other devices, surgical techniques, or anatomical terms. This approach was chosen to maximize specificity and to avoid retrieval of irrelevant records. Although the search strategy was intentionally limited to the trade name “Amandys” to maximize specificity, we acknowledge a potential, albeit low, risk of missing studies in which the implant may have been discussed without explicit use of the trade name.

The search was conducted without restrictions on study design. Only articles published in the English or French language were considered eligible. The reference lists of all included studies were manually screened to identify additional relevant publications.

Studies were eligible for inclusion if they:

- reported original clinical data on patients treated with Amandys® pyrocarbon interposition wrist arthroplasty;
- included adult patients with degenerative, post-traumatic, or inflammatory wrist joint destruction;
- reported at least one clinical, functional, or patient-reported outcome, complication, or revision/removal rate.

The following were excluded:

- biomechanical, cadaveric, or technical studies without clinical outcomes;
- conference abstracts without full-text availability;
- duplicate publications reporting identical outcome data.

Letters to the editor and short reports were included when they provided original clinical data and were analyzed qualitatively.

When multiple publications originated from the same institution with potentially overlapping patient cohorts, all studies were included for qualitative synthesis. To avoid double counting, the most comprehensive or most recent publication was preferentially considered for outcome aggregation, while earlier reports were used to describe early experience and complication patterns.

Study selection was performed independently by two reviewers, with discrepancies resolved by consensus.

All records retrieved from the database search were screened based on title and abstract. Full-text articles were subsequently reviewed to confirm eligibility according to the predefined criteria.

### 2.1 Data extraction

Data extracted from each included study comprised study design, number of treated wrists, patient characteristics, indications, duration of follow-up, clinical and functional outcomes, complications, and revision or implant removal rates.

### 2.2 Methodological quality assessment

The methodological quality of the included full-length clinical studies was assessed using the Coleman Methodology Score (CMS), a validated scoring system ranging from 0 to 100 for evaluating the methodological rigor of orthopaedic clinical studies.^9^ Letters to the editor and short reports were excluded from this assessment.

### 2.3 Data synthesis

Due to heterogeneity in study design, indications, outcome measures, and follow-up duration, a quantitative meta-analysis was not feasible. Results were therefore synthesized using a qualitative narrative approach.

## 3 Results

**Fig. 1:**
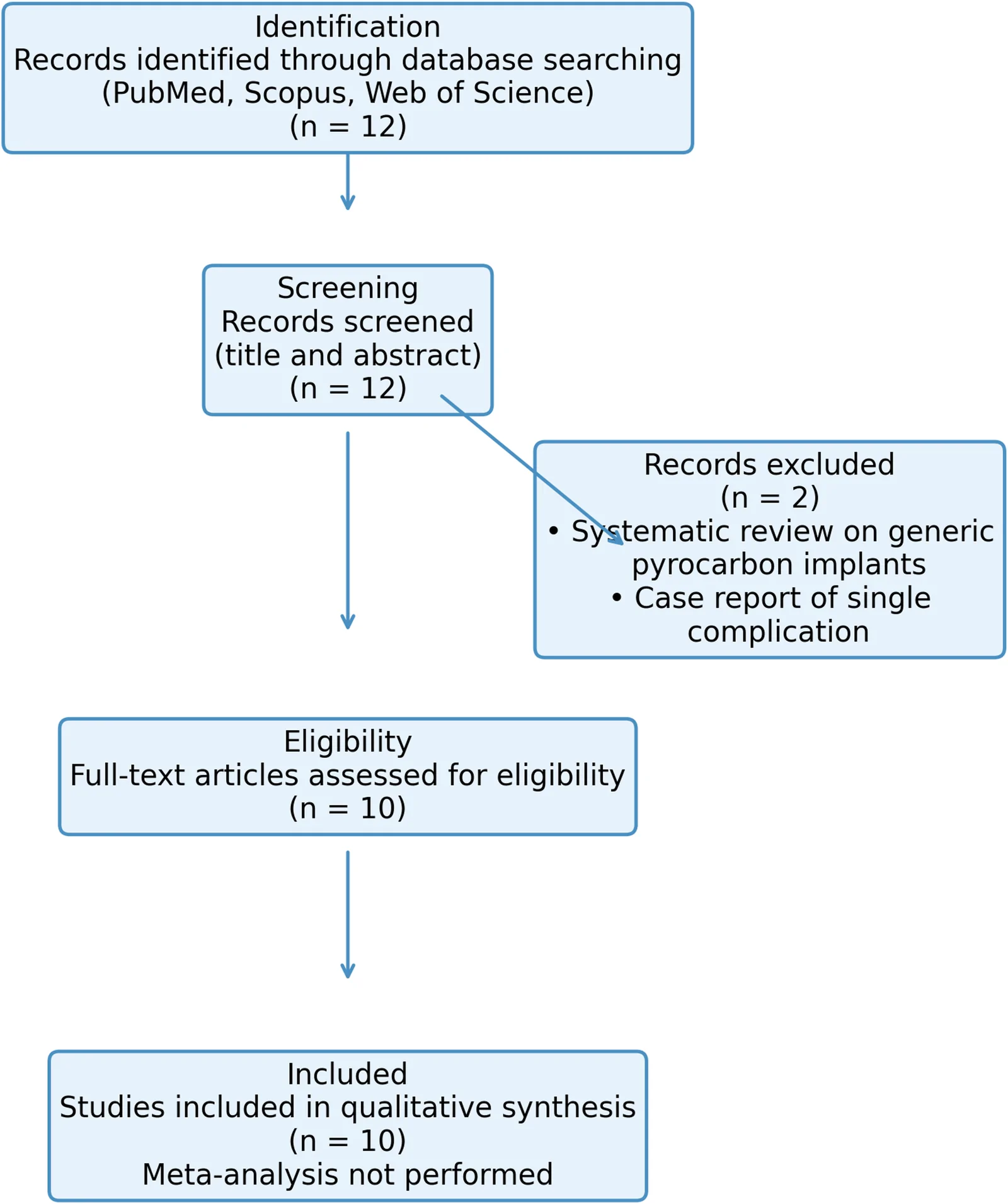
PRISMA flow diagram of study selection for the systematic review of Amandys® pyrocarbon interposition wrist arthroplasty.

Ten studies, reporting data on a total of 224 treated wrists, met the inclusion criteria and were included in the qualitative synthesis (Fig. 1). The main characteristics of the included studies are summarized in Table 1.

**Table 1:** Characteristics of the studies included in the systematic review of Amandys pyrocarbon interposition wrist arthroplasty. Follow-up is reported as mean duration; ranges are provided when available.

Study title	First author	Year	No. of patients/wrists	Follow-up	Study type
Amandys implant: Novel pyrocarbon arthroplasty for the wrist	Bellemère	2012	25 wrists	Mean 24 months (range 12-36)	Prospective case series
Pyrocarbon interposition wrist arthroplasty in the treatment of failed wrist procedures	Bellemère	2012	16 wrists	Mean 24 months (range 6-41)	Prospective case series (salvage)
Early results of a prospective study on the pyrocarbon Amandys for osteoarthritis of the wrist	Daruwalla	2012	6 wrists	Mean 16 months (range 8-28)	Prospective case series
Novel approach for post-traumatic panarthritis of the wrist using a pyrocarbon interposition arthroplasty (Amandys): Preliminary series	Pierrart	2012	11 wrists	Mean 11 months (range 6-21)	Retrospective case series
Results of interposition arthroplasty with the Amandys pyrocarbon implant in rheumatoid wrist at a mean 5 years' follow-up	Lestienne	2021	28 wrists	Mean 64 months (range 21-101)	Retrospective case series
Early clinical outcomes after Amandys interposition arthroplasty	Marie	2021	21 wrists	Mean 40,7 months (range 21-90)	Retrospective case series
A high incidence of early failure after Amandys wrist interposition arthroplasty among 13 cases	Gvozdenovic	2021	13 wrists	Mean 24 months (range 16-30)	Prospective short report
Medium-term outcomes for Amandys implant: a 5-year minimum follow-up of 63 cases	Tanwin	2022	63 wrists	Mean 84 months (range 60-132)	Prospective case series
Amandys pyrocarbon wrist replacement: early outcomes following 20 consecutive cases	Wharton	2023	20 wrists	Short-term	Letter with original data
Amandys versus four-corner fusion in patients aged over 50 years	Lozano	2024	21 Amandys wrists	Mean 60 months (range 36-72)	Retrospective comparative study

### 3.1 Demographics

Patient sex was reported in 9 of the 10 included studies, accounting for 204 patients. Overall, 59.3% were male (n = 121) and 40.7% were female (n = 83) 1, 2, 3, 4, 5, 6, 7, 8^,^^10^.

Sex distribution was not reported in one studies.^11^ Male patients predominated in degenerative and post-traumatic cohorts, whereas female patients were more frequently represented in inflammatory wrist disease, particularly in the dedicated rheumatoid arthritis series.^5^

Patient age was reported in most of the included studies, although not always as a mean value. Across the available data, the youngest reported patient was 20.3 years old and the oldest was 85 years old.^7^^,^^8^

A pooled mean age could be calculated from studies reporting a mean value, yielding an overall mean age of 56.4 years, based on 170 patients 1, 2, 3, 4, 5, 6, 7.

Age was not available as a mean for the Amandys® subgroup in the comparative study by Lozano et al.^10^ and was therefore not included in the pooled calculation. Age data were not reported in the letter by Wharton et al.^11^

### 3.2 Indications

Indications for Amandys® implantation were heterogeneously reported across studies. In series where indications were clearly defined and mutually exclusive, degenerative and post-traumatic wrist osteoarthritis represented the majority of cases, followed by salvage procedures after failed prior wrist surgery and inflammatory wrist disease.

In early and salvage series, indications included scapholunate advanced collapse (SLAC) and scaphoid nonunion advanced collapse (SNAC) wrists, distal radius malunion, failed partial wrist arthrodesis, failed proximal row carpectomy, and complications related to silicone implants 1, 2, 3. Inflammatory wrist disease was addressed in a dedicated cohort of 28 rheumatoid wrists^5^ and was also included within mixed-etiology series.^6^^,^^7^ In several studies, indications were reported using overlapping or hierarchical categories (e.g., post-traumatic osteoarthritis with sub-classification into SLAC, SNAC, or distal radius malunion), precluding reliable aggregation into pooled percentages without risk of double counting.^6^^,^^7^ Additionally, some studies did not provide a precise numerical breakdown of indications^4^^,^^11^ and in the comparative study by Lozano et al.^10^ indications were not separately reported for the Amandys® subgroup.

### 3.3 Immobilization and rehabilitation protocols

Postoperative immobilization protocols were reported in 7 of the 10 included studies. Among these, 2 studies described a short immobilization protocol, with wrist immobilization limited to approximately 2 weeks.^1^^,^^2^ In contrast, 5 studies reported a longer immobilization period, ranging from 4 to 6 weeks, typically using a splint or cast ^3^^,^^5^, ^6^, ^7^^,^^10^. The remaining 3 studies did not provide sufficient detail regarding postoperative immobilization duration.^4^^,^^8^^,^^11^ When reported, wrist mobilization was initiated either during the immobilization period or after splint removal. The use of supervised physiotherapy was mentioned in several studies, although the timing and structure of rehabilitation were not consistently specified.

### 3.4 Clinical outcomes

Clinical outcomes are summarized in Table 2. Across the included studies reporting quantitative data, pain reduction was consistently observed following Amandys® wrist arthroplasty. Considering all series reporting VAS or equivalent pain scores, mean or median pain values decreased from approximately 6–8 preoperatively to 0–3 postoperatively, corresponding to a clinically meaningful reduction in pain in the majority of treated wrists. Improvements were observed regardless of the pain scale used, including VAS, PRWE pain subscores, and pain numerical ratings.

**Table 2:** Clinical outcomes following Amandys pyrocarbon interposition wrist arthroplasty. VAS, visual analogue scale; ROM, range of motion; PRWE, Patient-Rated Wrist Evaluation; DASH, Disabilities of the Arm, Shoulder and Hand; NR, not reported; PEM, patient-evaluated measure.

Author, year	Pain (VAS or equivalent)	DASH/PRWE	ROM (flex–ext arc)	Strength
Bellemère 2012 (Chir Main)	PRWE pain/50: 34 → 18	PRWE total/100: 61 → 32; QuickDASH/100: 63 → 36	69° → 68°	Preop 15 kg → Postop 16 kg
Bellemère 2012 (J Wrist Surg – salvage)	PRWE pain/50: 34 → 19	PRWE: 57 → 33; QuickDASH: 59 → 39	67° → 68°	Preop 17 kg → Postop 19 kg
Daruwalla 2012	VAS: 8.2 → 3.3	DASH: 55 → 29	79° → 82°	14 kg (weaker than preop)
Pierrart 2012	Not available (pain reported qualitatively; no VAS)	PRWE: 64.14 → 38.35 QuickDASH: 61.61 → 42.88	78.6° → 71.5°	20.4 kg → 8.3 kg
Marie 2021	VAS: 6.8 → 3,1	PRWE: 37,9 QuickDASH: 29,6	Post op 69°	14,8 kg
Gvozdenovic 2021 (short report)	VAS (0–100): 66 → 29	qDASH: 50 → 24	68° → 69°	14 kg → 16 kg
Lestienne 2021 (RA)	VAS: 6 → 2	PRWE: 62 → 25 QuickDASH: 62 → 36	66° → 66°	10 kg → 17 kg
Tanwin 2022	VAS: 7 → 3	PRWE: 63 → 27 QuickDASH: 63 → 34	66° → 74°	12 kg → 20 kg
Wharton 2023 (letter)	VAS: 7.4 → 1	PEM: 56,4 → 34,8	Preop 61° → Postop 56°	Not available
Lozano 2024 (Amandys subgroup)	VAS: 7 → 0	QuickDASH: 70 → 24; PRWE: 68 → 26	Preop 76° → Postop 84°	14 kg → 19 kg

Note: Values are reported as presented in the original studies. Pain outcomes were variably reported (VAS, PRWE pain subscore, or pain score). Where mean flexion and extension were reported separately, the flexion–extension arc was calculated as their sum.

Patient-reported outcome measures showed a parallel improvement. Across studies reporting DASH, QuickDASH, or PRWE scores, functional outcomes improved in all series, with postoperative values consistently lower than baseline. When pooled descriptively, DASH/QuickDASH scores decreased from preoperative values typically above 55–70 to postoperative values in the range of 24–39, while PRWE total scores improved from approximately 57–68 preoperatively to 25–34 postoperatively, including both degenerative and inflammatory indications.

Wrist range of motion was generally preserved after surgery. In studies reporting pre- and postoperative values, the flexion–extension arc remained stable or showed modest improvement, with postoperative arcs most commonly ranging between 66° and 84°. Only one series reported a mild postoperative reduction in motion, whereas the remaining studies demonstrated maintenance or improvement of wrist mobility compared with baseline.^3^

Grip strength outcomes were heterogeneous but tended to improve when reported. In series providing quantitative data, grip strength increased from preoperative values of approximately 10–17 kg to postoperative values of 16–20 kg, whereas a reduction in strength was reported in a single early series.^3^ Overall, most studies described either improvement or preservation of grip strength relative to baseline, although reporting methods and units were inconsistent.

Across the included studies, pain scores and patient-reported outcome measures (VAS, DASH/QuickDASH, and PRWE) were the most consistently reported outcomes, whereas objective measures such as grip strength and detailed range-of-motion values were less uniformly available.

Data on patient occupation and return to work were not consistently reported across the included studies and could therefore not be synthesized.

### 3.5 Complications and revisions

Complications, reoperations, and implant removals following Amandys® wrist arthroplasty are summarized in Table 3. Considering all studies reporting complication data, a total of 41 complications were observed among 204 implanted wrists, corresponding to an overall complication rate of 20.1% (41/204).

**Table 3:** Complications, reoperations, and revision procedures after Amandys pyrocarbon interposition wrist arthroplasty.

Study	Implants (n)	Complications n (%)	Reoperations n (%)	Implant removals n (%)
Bellemère 2012 (Chir Main)	25	4 (16%)	2 (8%)	0 (0%)
Bellemère 2012 (JWS salvage)	16	2 (12.5%)	2 (12.5%)	0 (0%)
Daruwalla 2012	6	1 (16%)	1 (16%)	0 (0%)
Pierrart 2012	11	5 (45.5%)	4 (36.4%)	2 (18.2%)
Gvozdenovic 2021	13	7 (53.8%)	7 (53.8%)	4 (30.8%)
Marie 2021	21	6 (28.6%)	6 (28.6%)	6 (28.6%)
Lestienne 2021 (RA)	28	3 (10.7%)	3 (10.7%)	0 (0%)
Tanwin 2021	63	10 (15.9%)	10 (15.9%)	0 (0%)
Lozano 2024 (Amandys)	21	3 (14.3%)	1 (4.8%)	0 (0%)
Wharton 2023	NR	NR	NR	NR

NR = not reported. Percentages are calculated based on the number of implanted wrists in each study. Categories are not mutually exclusive.

Reoperations were required in 36 wrists, yielding a weighted reoperation rate of 17.6% (36/204). The most common reoperations were performed for mechanical instability, including implant rotation or dislocation, and typically consisted of capsuloligamentous retensioning, implant repositioning, or exchange to a different implant size.

Implant removal was performed in 12 wrists, corresponding to an overall explantation rate of 5.9% (12/204). Implant removal was predominantly indicated for early recurrent dislocation or persistent pain, and was mainly reported in the series by Marie et al. and Gvozdenovic et al.^7^^,^^8^ Following implant removal, patients underwent a variety of salvage procedures, including proximal row carpectomy with interposition, RCP prosthesis and total wrist arthrodesis.

Additional secondary surgeries not directly involving the implant, such as distal radioulnar joint procedures, tendon reconstruction, or pain-targeted interventions, were also reported in a small subset of patients. Reporting of complications at the individual patient level was heterogeneous. While some studies specified multiple complications or reoperations occurring in the same wrist, others reported only the total number of events, precluding reliable aggregation of multiple complications per patient across the entire cohort.

When reported, complications were predominantly early, occurring within the first 6 months after surgery and mainly related to mechanical instability. Late complications (>6 months) were less frequently described and more heterogeneous in nature.

### 3.6 CMS

The methodological quality of the included studies was moderate overall, with a mean Coleman Methodology Score of 57 (range 41–68). Most studies were retrospective case series, with higher scores observed in larger cohorts and comparative designs.

## 4 Discussion

This systematic review provides a comprehensive synthesis of clinical outcomes, complications, and revision patterns associated with Amandys® pyrocarbon interposition wrist arthroplasty, enabling a critical appraisal of its role among contemporary wrist salvage procedures.

Across the included studies, Amandys®arthroplasty was consistently associated with clinically meaningful pain reduction and improvement in patient-reported outcomes. Aggregated results demonstrated substantial decreases in pain scores and improvements in DASH, QuickDASH, and PRWE values, with postoperative scores comparable to those reported for established salvage procedures.^1^^,^^2^^,^^6^^,^^7^^,^^10^ For commonly used wrist-specific patient-reported outcome measures, such as DASH and PRWE, previously reported minimal clinically important difference (MCID) values range approximately between 10 and 15 points. In the present review, several studies reported improvements exceeding these thresholds, suggesting clinically meaningful benefit. However, due to heterogeneity in reporting, baseline severity, and follow-up duration, a formal MCID-based comparative analysis across studies was not feasible.Importantly, these benefits were observed across both degenerative and inflammatory indications. Some reported indications, such as failed partial wrist arthrodesis or failed proximal row carpectomy, may appear counterintuitive when considering the original description of the technique. In these cases, Amandys® arthroplasty should be interpreted as a secondary salvage procedure rather than a primary indication, reflecting complex clinical scenarios with limited reconstructive options. A formal correlation between these indications and failure rates could not be established due to heterogeneity in reporting.

Preservation of wrist motion represents a central rationale for interposition arthroplasty. In the present review, postoperative flexion–extension arcs were generally maintained or modestly improved compared with baseline, supporting the role of Amandys® as a motion-preserving option. However, improvements in grip strength were less consistent, suggesting that the primary benefit of the procedure lies in pain relief and functional use rather than power restoration. A formal comparison of outcomes between different indications, such as degenerative osteoarthritis and inflammatory wrist disease, was not possible due to heterogeneity in reporting and limited stratification of results by indication. The dedicated rheumatoid arthritis series reported clinically meaningful improvements in pain and function, but these outcomes cannot be directly compared with degenerative cohorts. Therefore, no definitive conclusion regarding superiority of outcomes by indication can be drawn.

Despite favorable clinical outcomes, the complication profile of Amandys® arthroplasty emerged as a critical limitation. When weighted by study size, approximately 20% of wrists experienced a complication, and nearly 18% required reoperation. The majority of secondary procedures were performed for early mechanical instability, including implant rotation, subluxation, or dislocation. From a temporal perspective, the majority of reported complications occurred early in the postoperative course and were predominantly mechanical or technical in nature, including implant instability, subluxation, or dislocation. Late complications were less frequently reported and were more heterogeneous, often related to progressive degenerative changes or persistent symptoms rather than implant-related failure.

Notably, most reoperations consisted of technically oriented interventions, such as capsuloligamentous retensioning, implant repositioning, or exchange to a different implant size, rather than immediate implant removal. Implant explantation and conversion to total wrist arthrodesis occurred less frequently, indicating that failure of Amandys® arthroplasty does not necessarily preclude further salvage options. These findings highlight that early mechanical stability, rather than intrinsic implant failure, represents the dominant challenge associated with this technique.

When technically feasible, proximal row carpectomy (PRC) remains the preferred motion-preserving surgical option. High-level evidence comparing PRC and four-corner fusion demonstrates similar pain relief and functional outcomes, with superior wrist motion and significantly lower complication and reoperation rates following PRC.^12^ In addition, PRC avoids implant-related complications, is technically reproducible, and is associated with lower overall costs, reinforcing its role as the first-line procedure whenever the articular surfaces of the capitate and radius are preserved.

In cases where PRC is contraindicated because of capitate degeneration, capitate resurfacing implants combined with PRC (RCPI) represent a logical extension of the same biomechanical concept. Systematic reviews and comparative studies have shown that PRC with RCPI provides range of motion and patient-reported outcomes comparable to PRC alone, with preservation of carpal height and potentially improved load transmission.^13^^,^^14^ However, the RCPI literature is characterized by heterogeneous reporting of complications, limiting robust statistical comparison. Recent series indicate that RCPI is not exempt from secondary surgery, including conversion to total wrist arthrodesis, suggesting that RCPI modifies rather than eliminates the risk of failure.^15^

Based on the present findings, Amandys® arthroplasty should not be considered a competing alternative to PRC or PRC-based solutions. Instead, it occupies a distinct and more selective role, particularly in patients with advanced degeneration of both the capitate and the radial articular surface, in whom PRC-based procedures are no longer feasible. In this context, the primary surgical alternatives are often total wrist arthrodesis or total wrist arthroplasty (TWA).

The bone-sparing nature of Amandys® arthroplasty represents a relevant advantage, as it preserves bone stock and facilitates potential conversion to total wrist arthrodesis compared with failed total wrist arthroplasty.

Contemporary fourth-generation TWA has demonstrated consistent pain relief and functional improvement, with pooled mid-term survivorship rates generally exceeding 90% at five years.^16^ However, TWA requires adequate bone stock and favorable soft-tissue conditions and is associated with its own spectrum of implant-related complications, which are variably reported in the literature. In contrast, Amandys® arthroplasty represents a bone-sparing, reversible option, preserving the possibility of later conversion to total wrist arthrodesis. Nevertheless, compared with TWA, Amandys® arthroplasty appears to be associated with higher rates of early mechanical complications and reoperations, emphasizing its role as a salvage rather than reconstructive procedure.

The overall methodological quality of the included studies was moderate, as reflected by a mean Coleman Methodology Score of approximately 57. Most studies were retrospective case series with heterogeneous indications, outcome measures, and follow-up durations. Definitions of complications and reoperations varied widely, and some outcomes were incompletely reported. These limitations precluded formal meta-analysis and necessitate cautious interpretation of pooled results. Furthermore, long-term survivorship data remain limited, particularly beyond ten years. It should be acknowledged that the Amandys® implant is no longer actively marketed, which may limit its current clinical use. Nevertheless, the present review remains relevant as it provides insights into outcomes, failure patterns, and decision-making principles applicable to interposition arthroplasty and wrist salvage procedures. Given the involvement of the same surgical teams in multiple publications, partial temporal overlap between cohorts cannot be completely excluded; however, none of the original studies explicitly reported duplicated patient populations.

## 5 Conclusion

Amandys® pyrocarbon interposition arthroplasty is associated with consistent pain relief, functional improvement, and preservation of wrist motion, but at the cost of a substantial and less predictable rate of complications and reoperations, predominantly related to early mechanical instability. When feasible, proximal row carpectomy remains the preferred motion-preserving procedure, offering comparable clinical outcomes with greater reliability, lower complication rates, and lower overall costs. In the presence of capitate degeneration, capitate resurfacing combined with PRC represents a logical alternative, while Amandys® arthroplasty should be regarded as a highly selective salvage option. In patients unsuitable for PRC-based solutions, Amandys® may be considered as a motion-preserving alternative to total wrist arthrodesis, with the understanding that careful patient selection and surgical expertise are critical to optimize outcomes.

## Informed consent

Not Needed.

## Ethical approval

Not needed.

## AI use statement

Portions of this manuscript, including language editing, reference formatting, and summarization of background literature, were assisted by a large language model (ChatGPT, OpenAI). All scientific content and conclusions have been critically reviewed and validated by the authors, who take full responsibility for the manuscript's integrity and accuracy.

## Funding

This research received no specific grant from any funding agency in the public, commercial, or not-for-profit sectors.

## Declaration of Competing interest

The authors declare that they have no known competing financial interests or personal relationships that could have appeared to influence the work reported in this paper.
